# Medical History from the Earliest Times

**Published:** 1893-07-15

**Authors:** E. T. Withington


### Medical History from the Earliest Times.

By E. T. Withington, M.A., M.B. Oxon.

### LXII.—THE SURGERY OF THE EIGHTEENTH CENTURY.

It is pleasant to turn from the speculative medicine of the eighteenth century to the contemporary surgery, which equalled in brilliance that of the age of the great anatomists, and was marked not only by important improvements in the art, but also by a decided rise of the profession in the social scale. As in the sixteenth century surgery found its basis in the new anatomy, so now it allied itself with the comparatively modern study of pathology, with the result that the surgeon, ceasing at length to be a mere mechanic, became a man of science. French surgeons, as befitted the countrymen of Paré and Franco, took a prominent part in the advance, but the place formerly occupied by Italy was now taken by our own country, though the work was carried on in different ways strikingly characteristic of the genius of the two nations. In France it centred in a State institution, the famous Royal Academy of Surgery, while in Britain it was made up of the scattered labours of private individuals, the most famous of whom, the immortal John Hunter, may be said to have comprised an academy in himself.

The most representative French surgeons were, in the past half of the century, Anel and Petit, and in the second half Desault and Chopart. Anel, who joined the army in early youth and attained the rank of surgeon major, gives us an interesting sketch of military medicine during the wars of Louis XIV. Educated surgeons were few, their place being supplied by "wound suckers," "some of whom are, or have been, soldiers, while others have never served, and are entirely ignorant of surgery. These all pretend to cure wounds by sucking them, after which they pour in a little oil, muttering certain charms, and then cover the whole with a compress shaped like a St. Andrew's cross." The charms, says Anel, are nonsense; the oil does neither harm nor good; the sucking, however, is sometimes of great use in removing blood clots and foreign bodies which prevent direct union. But it is disgusting and dangerous to do this with the mouth, and Anel therefore invented an ingenious suction syringe, which formed the germ of the complicated modern aspirators. His next achievement was the well-known operation for curing aneurism by ligature of the artery immediately above the sac, which he successfully performed on a monk at Rome, January 30th, 1710. Three years later he passed the first probe through the lachrymal duct of a living being, and invented a minute syringe to inject fluids into the sac, by which means he was able to cure cases of lachrymal abscess or mucocele without the use of knife or cautery.

J. L. Petit (1674-1750), like Anel, began as an army surgeon, and his experiences in that capacity doubtless aided him in the invention of the famous screw tourniquet, which preserves his name. He also improved the circular method of amputation by dividing the soft parts in two incisions instead of one, and he pointed out the great importance of removing all suspicious glands in the operation for cancer. Finally, he became director of the Academy of Surgery, which was founded in 1731 for the purpose of putting an end to the continual quarrels between the barbers, master surgeons, and physicians, and which, on its dissolution in 1792, had fairly earned the praise of being the most useful of all academies. Its "Memoirs" contain the best results of the Continental surgery of the age; it offered annual prizes for the investigation of disputed questions, and monarchs, such as Frederick the Great, sought its recommendation in appointing surgeons for themselves and their armies.

One of its greatest members was Pierre Desault (1744-95), whose name is still connected with a bandage for fractured collar-bone, but who did better service by the introduction of the gum-elastic catheter, the straight amputating knife, and the modern form of wire snare or écraseur, but above all, by the development of clinical instruction in surgery, in which he was aided by his friend Francis Chopart (1743-95), a practitioner known to every student from his operation on the foot.

The grand procession of British surgeons of the eighteenth century is opened by William Cheselden (1688-1752), of St. Thomas's, who was the first to manufacture an artificial pupil by making a slit in the iris (iridentomy), and discovered, independently of Petit, the method of circular amputation "by two incisions." But he owes his fame chiefly to his improvements in lithotomy, an operation which he rescued from the hands of the specialists and brought to such perfection that he is said to have gone through the whole process in 54 seconds, at the same time reducing the mortality to 7 per cent., a marvellous achievement in that age. Had he survived another year he might have broken his record, for in 1753 Cæsar Hawkins, of St. George's, invented the cutting gorget, a terrific implement now abandoned, but much valued at the time, since it enabled the surgeon to operate with greater rapidity and with less danger of wounding the rectum or pudic artery. The instrument was adopted and improved by the great Desault, who made it the subject of his inaugural thesis at the Academy.

To attempt to criticise the work of John Hunter (1728-95) in a paragraph would be equally absurd and presumptuous, and the reader must refer to the masterly descriptions given by the leaders of our profession in their annual orations. We need only notice here (1) the extent and variety of his researches, which give him a place in the history of British surgery analogous to the one held by the Academy in that of our neighbours; (2) the zeal with which he sought for principles without neglecting facts, thereby elevating surgery to the rank of a science, and accomplishing for that division of the healing art what the united labours of such men as Morgagni, Bichat, and Laennec afterwards did for medicine; and (3) his two chief special services to the art, the improvement of Anel's operation for aneurism, and his study of the nature of inflammation.

Percival Pott, of St. Bart.'s, and Benjamin Bell, of Edinburgh, though alike in the alliteration of their names, differed widely in their mode of work, for while the former devoted himself to the study of a few special diseases, on which he published monographs, the latter embraced the whole compass of the art, and his "System of Surgery" remained a recognised text-book far into the present century. But while the name of Pott will probably last as long as the swelling, the disease, and the fracture with which it is connected, the work of Bell is now utterly forgotten or confounded with those of his later namesakes, John and Charles.

As already noticed, it is characteristic of English surgery that it is not connected with any definite centre, and some of the greatest improvements in the art were made by provincial practitioners. Thus in 1678 Lowdham, of Exeter, had revived the method of amputating by flaps practised by the Greek surgeons of the past century; and about a hundred years later another important operation, that of excising diseased bones and joints, was restored almost simultaneously by White, of Manchester, and Park, of Liverpool, who may, therefore, be styled the fathers of conservative surgery.

The number of celebrated French and British surgeons in the last century is so great that many can only be named here, as for example La Peyronie, Louis, Ledran, Goulard, Sharp, of Guy's; the two Monro's, of Edinburgh; and Douglas, memorable through his researches on pelvic anatomy. Those of other nations may be briefly dismissed. Lawrence Heister was the Benjamin Bell of Germany. His "Institutes of Surgery" were translated into nearly all European languages, and almost rivalled in fame the medical "Institutes" of his master, Boerhaave. In Italy Antony Scarpa advanced the science of surgical anatomy, and, together with Cotugno, made important investigations into the structure of the internal ear. Finally Spain, after a long interval, produced in Antony Gimbernat a surgeon and anatomist, who might be mentioned in the same breath with her great men of old, Servetus, and Arnald of Villanova.

